# 

**Published:** 1905-06

**Authors:** 


### NOTICE.

The adoption by the State Medical Association of the journal method of publishing its transactions in lieu of the yearly volume in no manner affects either the status or the interests of the TEXAS MEDICAL JOURNAL ("The Red Back"). A misapprehension exists, created by a fool statement in the *Houston Chronicle* at the time, that the "Red Book" was "knocked out.". The "Red Back" was never the "official organ" of the Association, but for years, and up to date, it published full reports of its transactions, and everything relating to the Association that was of interest to members, including all legislative matters, and will *continue to do so.*

